# Genomic and epidemiological characteristics of SARS-CoV-2 in Africa

**DOI:** 10.1371/journal.pntd.0009335

**Published:** 2021-04-26

**Authors:** Jones Lamptey, Favour Oluwapelumi Oyelami, Michael Owusu, Bernard Nkrumah, Paul Oluwagbenga Idowu, Enoch Appiah Adu-Gyamfi, Armin Czika, Philip El-Duah, Richmond Yeboah, Augustina Sylverken, Oluwatayo Israel Olasunkanmi, Ellis Owusu-Dabo, Christian Drosten, Yaw Adu-Sarkodie

**Affiliations:** 1 Centre for Health System Strengthening (CfHSS), Kumasi, Ghana; 2 Department of Microbiology, School of Medical Sciences, Kwame Nkrumah University of Science and Technology (KNUST), Kumasi, Ghana; 3 Kumasi Centre for Collaborative Research in Tropical Medicine (KCCR), Kumasi, Ghana; 4 Department of Animal Science, Shanghai Jiao Tong University, Shanghai, People’s Republic of China; 5 African Field Epidemiology Network (AFENET), Accra, Ghana; 6 Shenzhen Institute of Advanced Sciences, Chinese Academy of Science, Shenzhen, China; 7 Department of Physiology, School of Medical Sciences, University of Cape Coast, Cape Coast, Ghana; 8 Faculty of Medicine, Transilvania University of Brasov, Brasov, Romania; 9 Institute of Virology, Charite University, Berlin, Germany; 10 Department of Theoretical and Applied Biology, Kwame Nkrumah University of Science and Technology (KNUST), Kumasi, Ghana; 11 Department of Microbiology, Harbin Medical University, Heilongjiang, People’s Republic of China; 12 School of Public Health, Kwame Nkrumah University of Science and Technology (KNUST), Kumasi, Ghana; Ben-Gurion University of the Negev, ISRAEL

## Abstract

Since late 2019, the coronavirus disease 2019 (COVID-19) outbreak, caused by SARS-CoV-2, has rapidly evolved to become a global pandemic. Each country was affected but with a varying number of infected cases and mortality rates. Africa was hit late by the pandemic but the number of cases rose sharply. In this study, we investigated 224 SARS-CoV-2 genome sequences from the Global Initiative on Sharing Avian Influenza Data (GISAID) in the early part of the outbreak, of which 69 were from Africa. We analyzed a total of 550 mutations by comparing them with the reference SARS-CoV-2 sequence from Wuhan. We classified the mutations observed based on country and region, and afterwards analyzed common and unique mutations on the African continent as a whole. Correlation analyses showed that the duo variants ORF1ab/RdRp 4715L and S protein 614G variants, which are strongly linked to fatality rate, were not significantly and positively correlated with fatality rates (r = -0.03757, P = 0.5331 and r = -0.2876, P = 0.6389, respectively), although increased number of cases correlated with number of deaths (r = 0.997, P = 0.0002). Furthermore, most cases in Africa were mainly imported from American and European countries, except one isolate with no mutation and was similar to the original isolate from Wuhan. Moreover, unique mutations specific to countries were identified in the early phase of the outbreak but these mutations were not regional-specific. There were common mutations in all isolates across the continent as well as similar isolate-specific mutations in different regions. Our findings suggest that mutation is rapid in SARS-CoV-2 in Africa and although these mutations spread across the continent, the duo variants could not possibly be the sole cause of COVID-19 deaths in Africa in the early phase of the outbreak.

## Author summary

Mutations frequently occur in SARS-CoV-2 and the mutant variants, ORF1ab/RdRp 4715L and S protein 614G have been strongly linked to increased infectivity and fatality in other countries. Although increased number of cases in Africa correlated positively with increased deaths, such deaths did not correlate positively with the duo variants, ORF1ab 4715L and S protein 614G in the early part of the outbreak. This could possibly be due to the younger aged population, lower comorbidity and divergent genetic factors.

## Introduction

The severe acute respiratory syndrome coronavirus 2 (SARS-CoV-2), which causes coronavirus disease 2019 (COVID-19), was first reported in Wuhan, China in late 2019, and has rapidly spread to become a global pandemic [1,2]. SARS-CoV-2 is an enveloped, non-segmented, positive sense, single-stranded RNA virus with a genome of 30 kilobases and has four structural proteins: spike (S), envelope (E), membrane (M) and nucleocapsid (N) [3,4]. In general, SARS-CoV-2 shares about 79.5% and 96% genome sequence homology with previously identified SARS-CoV and bat coronavirus, SL-CoV-RaTG13 respectively [5,6]. COVID-19, the acute respiratory disease caused by SARS-CoV-2, is self-resolving and can also be deadly. Severe disease onset might result in death due to massive alveolar damage and progressive respiratory failure [7]. Current epidemiological studies have shown that the mortality rate is higher in the older population and those with underlying medical conditions such as high blood pressure, renal problems, cancer, diabetes and obesity [8].

SARS-CoV-2-host cell interaction is mediated through an envelope-anchored spike protein on the virus, which facilitates the binding of the virus to the host cell receptor. The fusion of the virus with the host cell membrane enhances host cell entry [9]. A defined receptor-binding domain (RBD) of SARS-CoV-2 spike binds explicitly to the host cell receptor, angiotensin-converting enzyme 2 (ACE2), first in the lungs and then in multiple organs of the body [10]. Organ damage by SARS-CoV-2 is by direct attack through ACE2, and indirect attack by means of cytokine storm or blood clots [11–13]. Notably, the genome structure of SARS-CoV-2 follows the specific gene characteristic of known CoVs. The anterior 5′ end of the genome comprises Open reading frame (ORF) 1ab encoding ORF1ab polyproteins, while the 3′ end consists of genes encoding the structural proteins. Additionally, SARS-CoV-2 contains six (6) accessory proteins, encoded by ORF3a, ORF6, ORF7a, ORF7b, and ORF8 genes [14].

Studies have shown that mutations frequently occur in SARS-CoV-2 [15]. The efficacy of several antiviral drugs may be compromised by the changes caused by single nucleotide polymorphisms (SNPs), which lead to changes in amino acid sequence and ultimately in the functional viral protein(s) [16]. With the many challenges facing Africa’s health system, the characteristics of COVID-19 in Africa are yet to be fully elucidated. To clarify the genomic characteristics and mutations in SARS-CoV-2 in Africa to better understand future viral adaptability and characteristics on this continent, we collected and analyzed publicly accessible epidemiology and genome dataset during the early part of the outbreak. We also highlighted the various points of mutation and amino acid changes, pointing out the mutation types and effects on fatality through correlation analysis and further examined the difference between Africa and other western countries based on population genetics.

## Methods

For this study, a total of 224 publicly available genomes (69 from available 17 African countries) were randomly selected from the Global Initiative on Sharing Avian Influenza Data (GISAID) database (https://www.gisaid.org/) [17] up to August 17, 2020 for efficient processing. The downloaded sequences were aligned using the default settings of the web-based version of MAFFT (https://mafft.cbrc.jp/alignment/software/) (version 7), and NC_045512 sequence was used as reference genome [14,18,19]. After sequence alignment, the evolutionary history of the sequences was inferred using the maximum likelihood method implemented in IQ-TREE web-server (http://www.iqtree.org/) with the default settings [20]. The bootstrap consensus tree was inferred from 1000 replicates and was taken to represent the evolutionary history of the analyzed taxa [21]. The resulting tree was afterward displayed using the Interactive Tree Of Life (iTOL) v4 platform (https://itol.embl.de/) [22]. Branches corresponding to partitions reproduced in less than 50% bootstrap replicates were collapsed. There was a total of 29918 positions in the final dataset. The laboratory codes of the resulting 224 sequences used in this study are listed in the GISAID excel sheet.

To analyze the mutation points, we compared the differences between the aligned Africa sequences to the Wuhan NC_045512 reference genome using the diffseq function of EMBOSS explorer (http://emboss.sourceforge.net/) (the missing nucleotide variants or non-determined scaffold-N were not reported in the mutation analysis result). Afterward, the mutation points were functionally annotated. The gene and the resulting protein product location of each mutation were detected using the UCSC Genome Browser (https://genome.ucsc.edu/index.html). Based on this annotation, nucleotide sequences (or their variant position) within each annotated gene were identified using the NCBI SARS-CoV-2 nucleotide database (https://www.ncbi.nlm.nih.gov/nuccore/NC_045512.2?report=graph), and then translated to their respective amino acid codon variants using the Transeq function of emboss programme (https://www.ebi.ac.uk/Tools/st/emboss_transeq/). Common and unique mutation points were also analyzed and represented on Venn diagrams using the bioinformatics and evolutionary genomics web tool (http://bioinformatics.psb.ugent.be/webtools/Venn/).

## Statistics

All statistical analyses were carried out using the GraphPad Prism 7. Continuous variables were compared using the Student’s t test. Fisher’s exact test was used to analyze differences between cumulative cases of SARS-CoV-2 and fatality rate. Pearson’s correlation was used to evaluate correlations among cumulative cases, total death, mutant variants and fatality rates. P: p-value; r: Pearson correlation coefficient. P< 0.05 was considered statistically significant. A positive r value was taken as a positive correlation and vice versa but statistically interpreted based on the p-value.

## Results

### Epidemiology of COVID-19 in Africa

**Fig 1:**
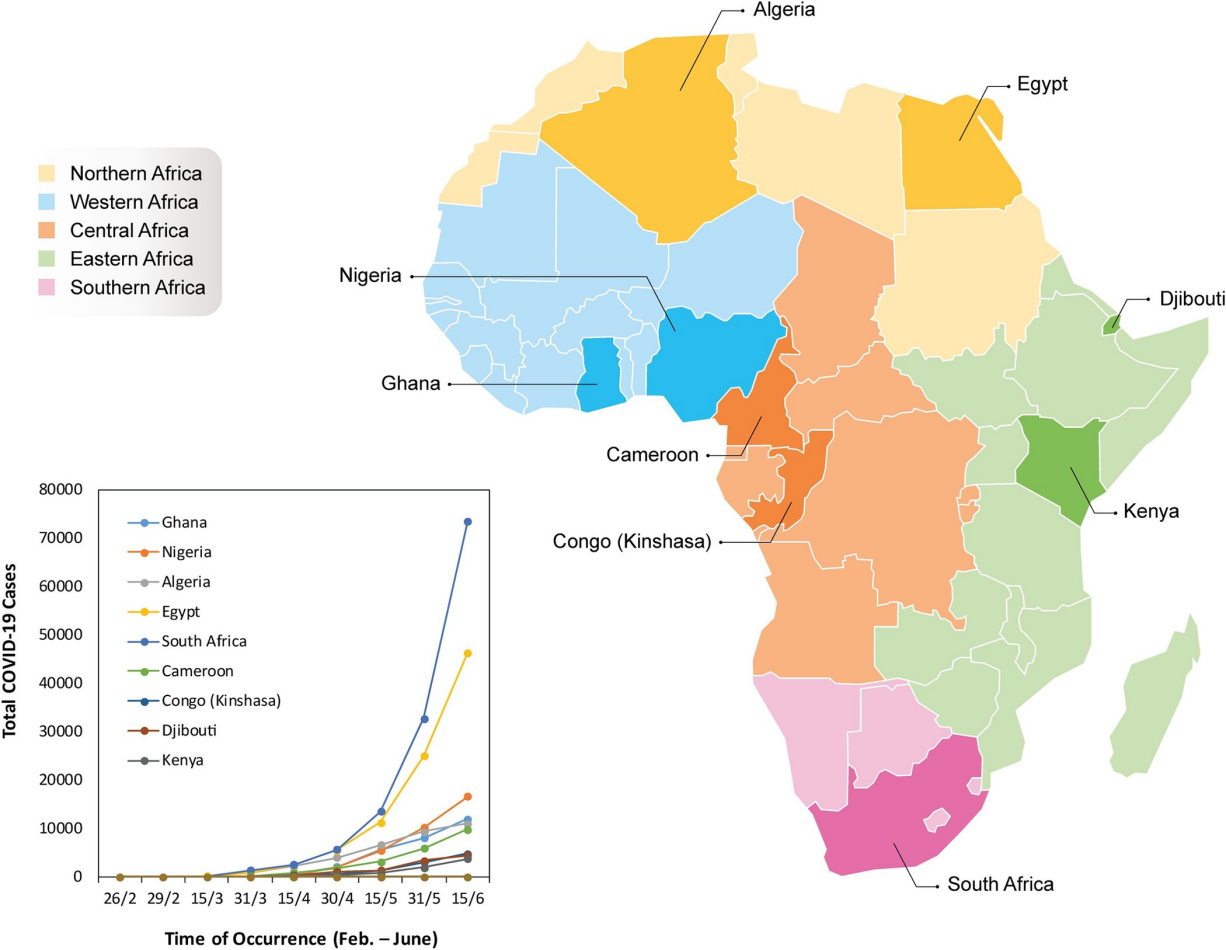
Epidemiological characteristics of COVID-19 in Africa. Cumulative cases of COVID-19 in Africa. Countries indicated on the map represent those with highest number of cases in each five regions (West, East, Central, Southern and North Africa) as of June 15, 2020. Highlighted countries represent countries with highest number of cases in the five regions at the early phase of COVID-19 in Africa. **The figure contains information from OpenStreetMap and OpenStreetMap Foundation, which is made available under the Open Database License**.

**Fig 2:**
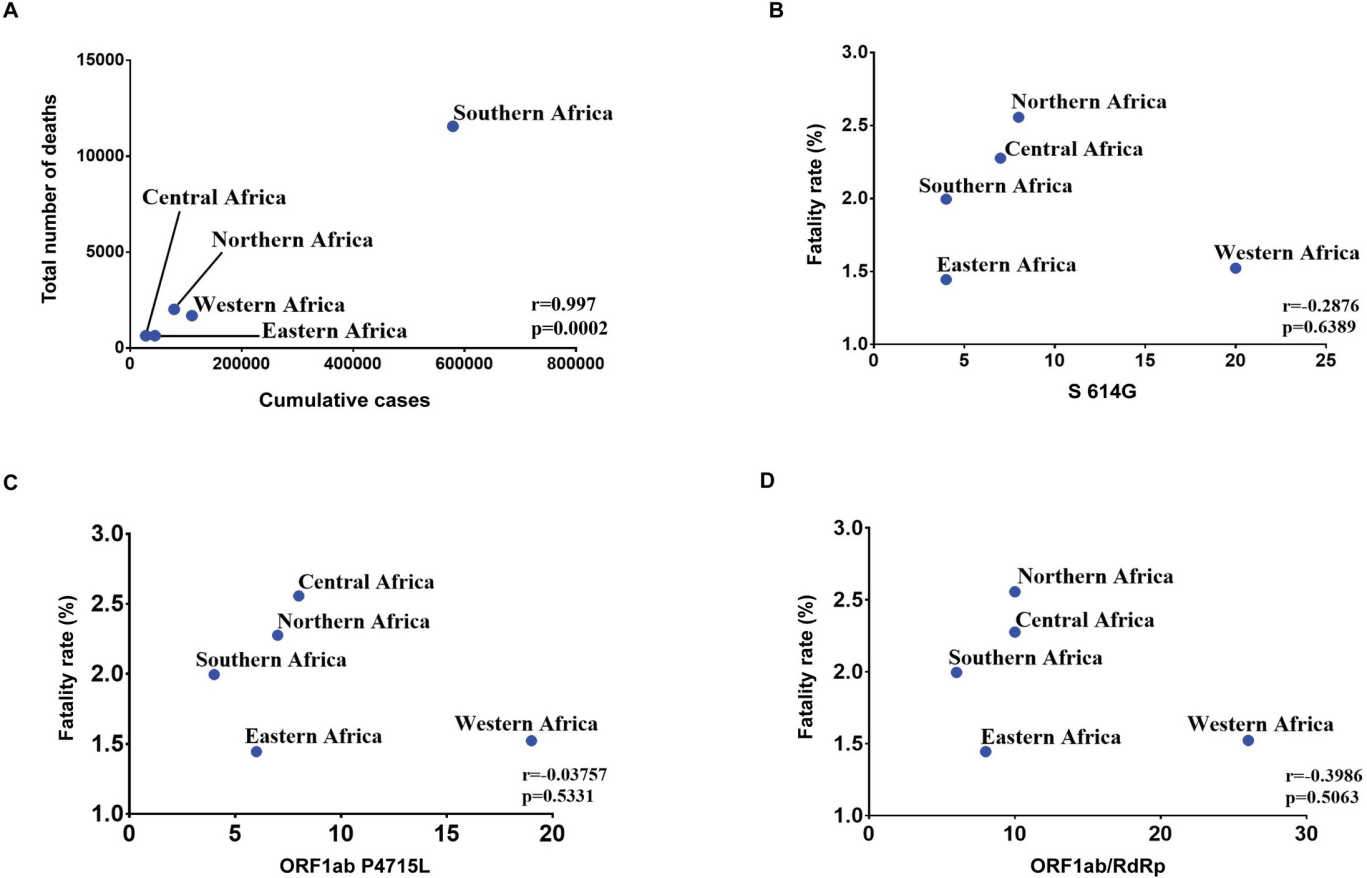
Correlation analysis of COVID-19 cases and mutant variants with fatality rate. Correlation analysis of cumulative cases with number of deaths (a); variant frequencies of SARS-CoV-2’ S 614G with fatality rates of COVID-19 among the five regions of Africa (b); ORF1ab P4715L with fatality rates of COVID-19 among the five regions of Africa (c); ORF1ab/RdRp with fatality rates of COVID-19 among the five regions of Africa (d). Pearson’s correlation coefficients (r) and p values were calculated.

To determine the trend in rise in cases in Africa, we collected data from the Johns Hopkins Center for Systems Science and Engineering (Baltimore, MD, USA) from the first detected cases in each African country up to mid-August, 2020. Our data show that the pattern of spread of COVID-19 in Africa was similar to that of other western countries. Initially, there were one or very few cases, which then rapidly surged, by at least 8-fold by the end of April, possibly due to asymptomatic human-to-human transmission (Fig 1). By mid-May, there was an increase from 1.2-fold to 2.7-fold in some countries in Africa. The statistical analyses (Fig 2) showed that the rise in number of cases correlated positively with the total number of deaths (r = 0.997; p = 0.002), but this was not significant after correlating regional distribution of COVID-19 cases with fatality rate (r = 0.6804; p = 0.2062). Furthermore, there was no correlation between mutant variants (S 614G, ORF1ab P4715; ORF1ab RdRp) and fatality rate (Fig 2) as reported in the western countries.

### Phylogenetic network analysis of SARS-CoV-2 in Africa

**Fig 3:**
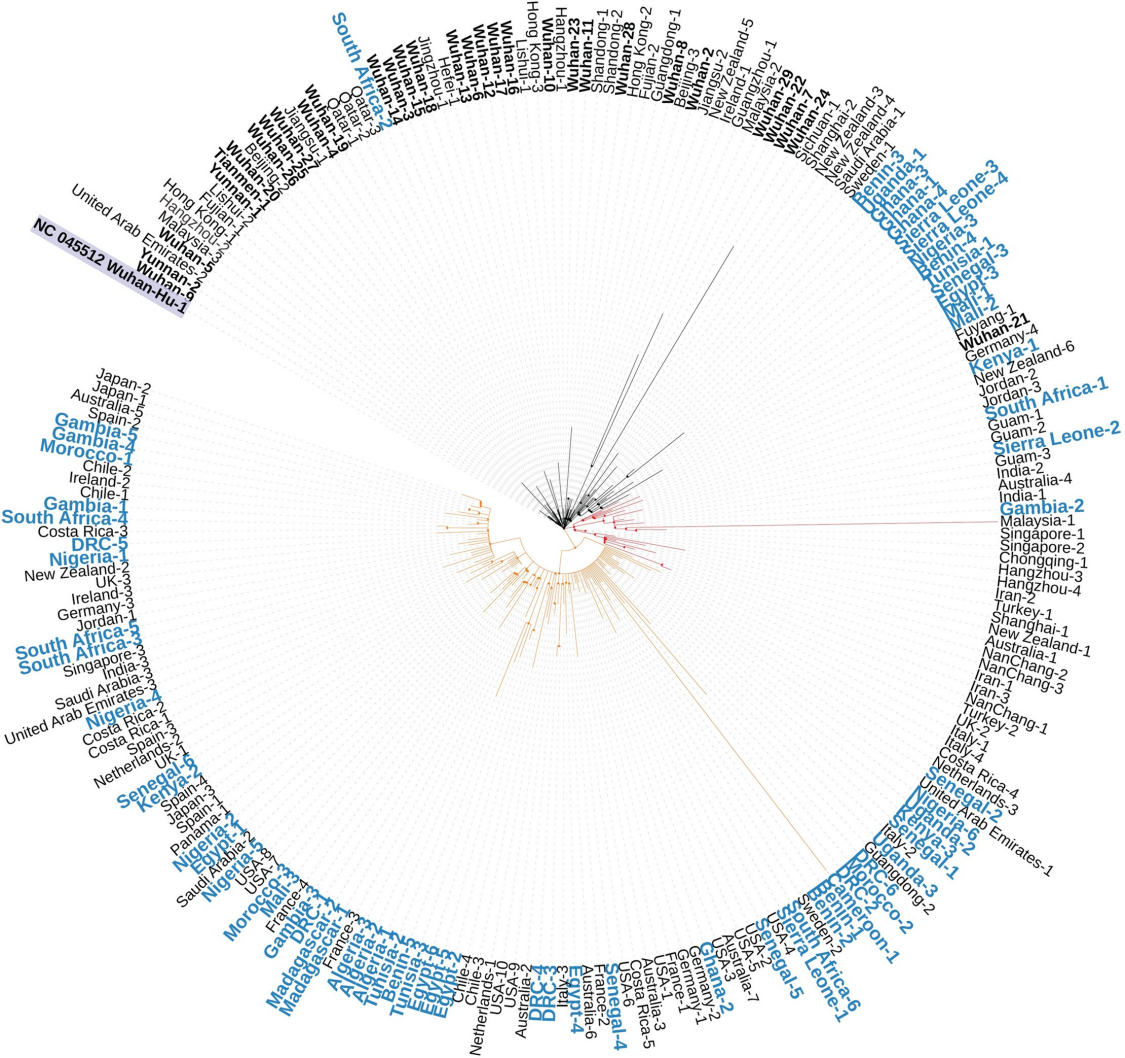
Phylogeny analysis of SARS-CoV-2 in Africa. Phylogeny analysis SARS-CoV-2 using 224 genome sequences form GISAID and NC_045512 reference genome from NCBI. Phylogenetic tree is divided into clades, and all clades are further divided into sub-groups. There were three clades formed: one from the highlighted NC 045512 Wuhan-Hu-1 isolate to Mali-2, another from Fuyang-1 (an isolate from China) to Iran-3, and the last from Nanchang -1 (from China) to Japan-2. Labels in blue represent African countries.

We determined the relationship among Africa SARS-CoV-2 isolates based on published genome sequences from the region. We also examined their evolutionary relatedness with other isolates from across the globe. As shown in Fig 3, most cases in the African Region were related to those from Europe and America, farther away from most of the Wuhan isolates, suggesting that the viral type transmitted to Africa are the mutated form of the original SARS-CoV-2 virus from Wuhan which first spread to Europe, America, and the Oceania. Interestingly, we found that among the African isolates, only the South Africa-2 isolate showed the closest relationship with the original SARS-CoV-2 virus from Wuhan and other viral isolate from China and Asia as its nucleotide sequence was the same as that of the original Wuhan viral sequence, suggesting that this case might have been from a patient who got infected at Wuhan, China, before travelling to South Africa. Other Africa isolate closer to the Wuhan, China, and Asia viral cases, are those between Benin-3 and Mali-2 isolate (Fig 3). Generally, there were three clades formed: one from the highlighted NC 045512 Wuhan-Hu-1 isolate to Mali-2, another from Fuyang-1 (an isolate from China) to Iran-3, and the last from Nanchang -1 (from China) to Japan-2.

### Nucleotide variations and amino acid change in SARS-CoV-2 in Africa

Since most African countries have relatively higher temperature range, we sought to determine the mutations and the general behavioral pattern of SARS-CoV-2 in this Region. The mutation points are presented in the supplementary sheet (S1 Table). Furthermore, common and unique mutations among the isolates have been summarized on the Venn diagram (S1 Fig). In all, a total of 550 nucleotide variations were used in the analysis. Computation of these variations yielded 307 non-synonymous, 172 synonymous, 69 non-coding and 1 non-mutated isolate. The 307 non-synonymous mutations occurred in S gene (No. = 57; 10.36%), N gene (No. = 30; 5.45%), E gene (No. = 5; 1.09%), M gene (No. = 4; 0.72%), ORF1ab (No. = 153; 27.82%; with 3C-like proteinase 0.18%; Exon 1.82%; helicase 0.18%; NSPs 14.36%; Pol 0.72%; pp1ab 0.72% and RdRp 9.82%)), ORF3a (No. = 35; 6.36%), ORF6 (No. = 2; 0.36%), ORF7a (No. = 4; 0.72%) and ORF8 (No. = 17; 3.09%). Generally, the synonymous mutation in the S gene was 18.63% and had mostly occurred at 23403A>G with the amino acid change, D614G (84.2%) and running through most countries in Africa. Out of the 30 non-synonymous mutations in the nucleocapsid, 33.3% were deletion and insertion at 28881_28883delinsAAC. The most common non-synonymous mutations were found in the ORF and S genes. Normally, cleavage of ORF1ab yields several nonstructural proteins (NSP1-NSP16). Among the NSP’s analyzed in this study, NSP3 had more variants, and seen in all countries studied. In terms of base changes, the most frequently observed was C>T (No. = 117/307; 38.11%).

### In-country genomic characteristics of SARS-CoV-2 in Africa

Based on the variations in nucleotide and amino acid changes, we next sought to determine the shared variations in the various isolates within countries in Africa (S1 Fig and S1 Table). Our analysis showed that three isolates (1, 3 & 4) out of four isolates from Ghana shared five common mutation points (29742 G>A, 28878 G>A, 24370 C>T, 8782 C>T, 28144 T>C) while mutation in ORF1ab/NSP2 (2306 C>T) occurred in two isolates, 1 & 4-. Uniquely, isolate 2 (with 8 mutations occurring in the ORF1ab/NSP2, ORF1ab/NSP3, ORF1ab/RdRp and S) shared no common mutation with the other isolates from Ghana. Isolates 3, 4, 5 & 6 from South Africa shared 14408 C>T nucleotide variant, with isolates 4, 5 & 6 having amino acid change D614G, and L3606F in isolates 1 & 4. Apart from isolate 2 from South Africa which had a nucleotide sequence same as that of the original Wuhan virus which could imply a possible infection during the early phase of the outbreak from Wuhan, all the other 5 isolates from South Africa had unique mutations, with some isolates having deletions and insertions in the Nucleocapsid region. Generally, our results showed that isolates within each country had some common nucleotide variations with changes in amino acids, as well as some unique mutations.

### Regional distribution of SARS-CoV-2 mutation across Africa

Based on the unique and common mutations in each country, we determined if these common and unique mutations were distributed across Africa. Due to travel restrictions implemented based on the initial few cases detected, the distribution of these mutated strains could rule-out the possibility of other imported cases and further enhance our understanding of the general behavior of SARS-CoV-2 across Africa. First, we analyzed these mutation points on regional bases (Northern, Southern, Eastern, Western and Central Africa) and then in all countries in Africa (S1 and S4 Tables). The regional analysis revealed that, from Central Africa, the isolate from Cameroon (isolate 1) and Democratic Republic of Congo (DRC; isolates 1–6) shared common nucleotide variations 14408 C>T, with all isolates from DRC having the amino acid change D614G (S4 Table). Isolates from Northern African countries such as Algeria (1–3), Egypt (1, 2 & 4) Morocco (1–3) and Tunisia (2,3) had common non-synonymous mutations in the ORF1ab/RdRp region. Our results also showed mutation in the S protein, with the amino acid change D614G in isolates from Algeria (1–3), Egypt (1, 2, 4), Morocco (1–3) and Tunisia (3). Isolate 1 & 2 from Morocco had deletions and insertions and multiple mutations in the ORF1ab/NSP15, respectively. In all these regions, some isolates had unique mutations although they shared common mutation points with other isolates.

Nucleotide variation analysis from the onset of the outbreak in Africa to mid-May revealed that isolates from majority of the African countries had unique mutation points. Contrary to this and the regional analysis, computational analyses of all mutation points revealed that no unique mutation was associated with any particular isolate. All isolates across the continent shared common mutation points. Thus, mutation points that were unique to a particular isolate in some parts of the continent were found in other parts of the continent as well. This probably shows the general behavior and adaptability of the virus as community transmission progressed.

## Discussion

Although most countries had reported cases of COVID-19 as of early April 2020, the reported number of COVID-19 cases was highest in the U.S., followed by Spain, Italy, Germany, France and China. Cases in Africa also rose rapidly in April, and as at July 22, 2020, South Africa had the highest number of cases (394,948) in the Southern part of Africa, followed by Egypt (89,745), Morocco (17,962) and Algeria (24,872) in the Northern part; Ghana (29,672) and Nigeria (38,344) in the Western part; Cameroon (16,522) in the Central part; and Djibouti (5030) in the Eastern part. The rise in cases could be due to population size and air traffic in most parts of the continent [23]. Generally, the limited healthcare system and testing capacities coupled with the limited human resources in most of the African countries could have also contributed to the increased cases, due to delay in prompt reporting. Increase in cases among different age groups and people with underlying medical conditions further increased the case fatality rate (CFR) of COVID-19 generally [24]. Reports showed that CFR was higher in elderly people and people with underlying medical conditions. For instance, as at mid-May, Italy with about 23% of elderly population, recorded CFR of 14.1%, Spain (230,698 cases, 27,563 deaths, CFR 11.9%), US (1,456,029 cases, 88,211 deaths, CFR 6.1%) as against South Africa (13,524 cases, 247 deaths, CFR 1.8%), Nigeria (5450 cases, 171 deaths, CFR 3.1%), Ghana (5,638 cases, 28 deaths, CFR 0.5%) and China (84,038 cases, 4,637 deaths, CFR 5.2% as against previously reported 1.2%) [25]. Most of the CFR decreased as at mid-August while other countries continued to have high CFR: Italy (13.94%), Spain (8.22%), US (2.0%), Nigeria (2.01%), Ghana (0.55%), China (5.25%), Algeria (3.59%), Egypt (5.33%) and Djibouti (1.1%). As seen in other countries, increased number of cases led to increase CFR [26]. Amidst the initial challenges with testing capacity in Africa [27] and subsequent improved surveillance and case finding [28], we sought to determine if similar pattern of detected and increased cases could increase fatality in Africa. Our analysis showed that increased number of cases correlated positively with increased number of deaths but this correlation was not significant when we clustered COVID-19 cases on regional basis. This is in contrast to a study where fatality correlated with regional distributions in the US [15]. Furthermore, we determined if the increased death from the increased cases could be due to any of the mutant variants of SARS-CoV-2. There was no correlation in this analysis which is in contrast to an earlier study in the US [15]. This difference could partly be explained by age and comorbidity. The frequency of Africa’s populations aged ≥60 years per thousand individuals is remarkably lower (frequencies 3.385, 2.686 and 3.528 for Ghana, Kenya and Ethiopia respectively) compared to western countries (frequencies 23.021, 21.228 and 18.517 for Italy, France and USA, respectively) [29]. According to the United Nations, 20% of Africa’s population consists of youth aged 15–24 (226 million in the year 2015). Adding to this number those below the age of 35years, increases the younger population of Africa to three quarters of its population [29]. Since age influences immunity to pathogens, the young African population could generate protective cell-mediated adaptive immunity which could decrease disease severity compared to older population in the western world [29,30]. Increasing age leads to immunosenescence which in turn is associated with many disorders such as cardiovascular diseases, metabolic diseases, neurological diseases, articular damage and cancers [31,32]. Higher fatality has been associated with COVID-19 patients with these comorbidities [24,33]. The lower mortality rate in Africa due to COVID-19 has therefore been associated with a higher younger population and lower comorbidity in many recent studies [34].

One major characteristic of SARS-CoV-2 is the frequent mutation and ease of spread of these mutated isolates. Our analysis showed 57 isolates with non-synonymous mutation in the S protein of SARS-CoV-2, all occurring at 23403 A>G (D614G) in 48 isolates (84.2%). Structural analyses from other studies indicated S protein having a D614G substitution is located on the surface of the virus and interacts with ACE2 [15]. The major determinant of host cell tropism in coronaviruses is the S protein [35], making it the most important site for amino acid mutation and enhancing immune evasion [36]. Both SARS-CoV and SARS-CoV-2 target the ACE2 receptor for cell entry through the protease-mediated cell-surface pathway and endosomal pathway. Priming of S protein by the help of cellular proteases, including furin, transmembrane protease serine 2 (TMPRSS2), cathepsin (Cat) B/L and elastase-2 enhance cell entry [35]. Recent studies showed that the elastase-2 cleavage site is a novel site in the S-G614 protein of SARS-CoV-2 variant [18,37]. It has been previously reported that SARS-CoV-2 isolates harboring the D614G mutation effectively cleaves S 614G with the help of elastase-2, thereby enhancing viral entry into 293T-ACE2 cells [18]. The D614G mutation is a common mutation in about 84.2% of isolates in Africa, and it is found in all isolates from Algeria, Morocco and the Democratic Republic of Congo (DRC). Studies have shown that the D614G mutation also enhances viral infectivity and transmissibility [38–41] and together with its highly linked variant, ORF1ab 4715L (essential for viral RNA replication) correlates positively with the fatality rate of COVID-19 [15]. The S D614G is located in the epitope sequences of S606-615, NQVAVLYQDV, and S612-620, YQDVNCTEV. Both wild-type and mutated epitopes have similar binding affinities for HLA-A*02:06. Similarly, ORF1ab P4715L is located in Nsp12 and in the epitope sequences of ORF1ab 4713–4721, FPPTSFGPL, ORF1ab 4713–4722, FPPTSFGPLV, and ORF1ab 4715–4724, PTSFGPLVRK, which respectively have strong binding affinities of 44, 41, and 45 nM to HLA-B*07:02, HLA-B*54:01, and HLA-A*11:01. HLA genotypes have been associated with susceptibility or resistance to SARS-CoV and MERS, including HLA-B*07:03, HLA-B*46:01, HLA-C*08:01, HLA-C*15:02, HLA-DRB1*03:01, HLA-DRB1*11:01, and HLA-DRB1*12:02 [15] Contrarily, other studies showed that, with regards to SARS-CoV-2 and the duo variants (ORF1ab P4715L and S D614G), populations with relatively high HLA-A*11:01, HLA-A*02:06, and HLA-B*54:01 alleles showed lower confirmed cases and fatality rates although these correlated data were not statistically significant as evidenced by a multiple regression analysis. This implies that individuals with HLA-A*11:01, HLA-A*02:06, or HLA-B*54:01 might be protected from SARS-CoV-2 infection [15]. Our results revealed that mutation in ORF1ab 4715L occurs in about 11.5% of detected cases and mutation in ORF3a occurred in about 6.8%. Although SARS-CoV-2 induces apoptosis in infected cells, pro-apoptotic activity of ORF3a in SARS-CoV-2 is significantly lower than that of SARS-CoV [42]. On one hand, it might be easy to assume that the mutation in the duo variants could contribute to increased number of cases in the Region, but on the other hand, mutation in some of the core genes limited the fatality rate in Africa, but this warrants further clinical and experimental studies. The difference in infectivity and fatality rate among Africa and other western countries despite the detection of these mutations in Africa could be explained on the basis of population genetics. Major risk variants associated with SARS-CoV-2 infection involve genes aiding viral entry (ACE2, TMPRSS2 and Furin), cytokine production (IFN-γ and IL4), and immune responses (ICAM3, CCL2, CCL5, AHSG, MBL, and CD209). A recent study showed that African have a genetic predisposition for lower expression levels of both ACE2 and TMPRSS2 genes and all risk variants more commonly detected in Europeans (TMPRSS2, Furin, ICAM3, and IFN-γ), were significantly lower among Africans [43]. Furthermore, since African descent have a lower response to ACE inhibitors compared to calcium blockers and β-adrenergic blocker anti-hypertensives [44], this decreased response could potentially contribute to the low COVID-19 prevalence and fatality in Africa. Moreover, population genetics studies showed great differences in the immune response between Africans and Europeans, in relation to genes necessary for inflammatory and antiviral responses [45]. The fold difference in allelic frequencies between populations observed in rs2280788 (CCL5 gene) was found in 9.5% of Eastern Asian population compared to 0.3% only among Africans. Similarly, rs1800450 in the mannose binding lectin (MBL) gene associated with SARS-CoV-2 susceptibility was found in 22% of Americans compared to only 1.36% of Africans [29]. Moreover, in the 1000 Genomes Project, the Neanderthal-derived haplotypes, which increased susceptibility to SARS detected in other races were almost completely absent from Africa population. The allelic frequencies of the Neanderthal core haplotype in south Asia are 30%, 8% in Europe, 4% among admixed Americans and at lower allele frequencies in east Asia. The study also reported that about 50% of people in South Asia carry at least one copy of the risk haplotype, whiles its carrier frequencies in Europeans and admixed Americans are about 16% and 9% respectively, supporting the hypothesis that gene flow from Neanderthals into African populations was limited and an additional explanation of the population difference in COVID-19 fatality rate [46].

Many studies have also reported the function of ORFs and ACE2 genes in the pathogenesis of COVID-19 [14]. ORF1ab is of high interest, as it occupies two-thirds of the genome of coronaviruses and encodes a replicase polyprotein from ORF1a and ORF1b. The ORF1ab encodes Papain-like protease (PLpro) and 3C-like protease (3CLpro) and is cleaved into 15–16 non-structural proteins (NSP1-NSP16) at consensus cleavage sites. Some of these nsps encode proteins that are important to the biology of RNA viruses, such as PLpro (nsp3), 3CLpro (nsp5) and RdRP (nsp12). The RdRp is required by most RNA viruses (except retroviruses) for replication and transcription of the viral genome [3], making it essential for their survival, and as it is a conserved protein within RNA viruses, it could serve as a potent candidate for further structural studies and antiviral drug development [16].

Our results further showed a non-synonymous mutation in the ORF8 of SARS-CoV-2 occurring at 28144T>C with the amino acid change L84S, except for three mutations at 28116G>A (D75N), 28219T>C (L109S) and 27942C>T (H17Y). ORF8 is a hotspot of mutation in CoVs but this mutation is associated with less systemic proinflammatory cytokine and milder clinical symptoms [47,48]. Although previous studies showed that ORF8 protein does not contain a known useful motif or region, a recent study showed that SARS-CoV-2 rapidly replicates in vivo without antiviral immune monitoring and this is crucial for immune evasion [49]. Studies also showed that the ORF8 of SARS-CoV-2 directly interacts with MHC-I molecules and significantly downregulates their surface expression on HEK293T cells, in contrast to ORF8a and ORF8b of SARS-CoV [47]. This is possible through selectively targeted degradation of MHC-I molecules via autophagy-dependent mechanism, thereby disrupting antigen presentation. This study also showed that exposure of healthy human donor-derived cytotoxic T lymphocytes (CTLs) sensitized to the SARS-CoV-2 epitope SSp-1, to autologous dendritic cells pre-pulsed with SSp-1, inefficiently eliminates ORF8-expressing HEK293T cells [49,50]. Thus, ORF8 protein disrupts antigen presentation by reducing the recognition and the elimination of virus-infected cells by CTLs. MHC-I allelic variability is associated with susceptibility and severity of SARS-CoV-2 and alleles able to efficiently present SARS-CoV-2 peptides are associated with milder COVID-19 fatality rate [51]. In Africa, the lower fatality rate of COVID-19 has been linked to the prevalence of different HLA alleles including the HLA-B*46:01 and HLA-B*15:03. Individuals with the HLA-B*46:01 allele could be especially vulnerable to COVID-19, as with SARS, since this allele has the fewest possible binding peptides for SARS-CoV-2. More prevalent in the African Region and other countries endemic for malaria is the HLA-B*15:03. HLAB*1503 demonstrates the greatest ability to present highly conserved SARS-CoV-2 peptides shared among common human coronaviruses, indicating that this allele may allow cross-protective T-cell dependent immunity [52]. Studies have shown that the interaction between HLA alleles and viruses could be a complex one. For instance, HLA-B27 prevalent in malaria-endemic is thought to confer susceptibility to malaria while conferring resistance to hepatitis C virus (HCV) and human immunodeficiency virus (HIV) [53–55]. The low prevalence of this HLA allele has been hypothesized to contribute to the lower cases reported in Africa, although a recent study showed a correlation between HLA-DRB1*15:01, -DQB1*06:02, and -B*27:07 and severe COVID-19 outcome. This correlation has been attributed to small sample size use in the study [51].

Our study has some limitations: First, since the sequences used in this study were downloaded from the GISAID database, we were unable to provide individual COVID-19 patients’ primary data where we could correlate some of the findings in this study with patients’ demographic characteristics and genetics. Some studies have attributed the lower number of cases in Africa to the younger population and genetic background and it would be enlightening to statistically determine if age, socioeconomic background affects viral mutation. Other studies have determined the genetic background of Africa population with COVID-19 outcome but these studies are few. We could only interpolate the findings in this study with available data from other studies. Secondly, the sequences used in this study were from the early phase of the COVID-19 outbreak in Africa and hence the small sample size in our study. We strongly believe that newer sequences uploaded might have some mutation different from the ones reported in this study and we encourage future studies to investigate these new mutations to elucidate the general adaptability of SARS-CoV-2 in Africa.

## Conclusion

In conclusion, we showed that, most cases of SARS-CoV-2 in Africa were related to the American and European isolates. CFR in most African countries in the early phase of COVID-19 outbreak was generally low although increase in cases correlated with number of deaths. Furthermore, we showed that nucleotide variations with corresponding amino acid changes occurred in SARS-CoV-2 that could contribute to viral pathogenesis and virulence, but mutation in the duo variants (ORF1ab P4715L and S 614G) did not correlate positively with fatality rate in Africa at the early part of the outbreak in Africa. Moreover, unique mutations specific to countries were identified in the early phase of the outbreak in Africa but these mutations were not regional-specific, showing the general behavior and adaptability of the virus in many African countries. Lastly, there were common mutations in all isolates across the continent as well as similar isolate-specific mutations in different regions. It remains to be elucidated if future mutations in SARS-CoV-2 could be detrimental to the African continent or if genetic and host factors highlighted by many studies could contribute to immune protection, although these factors would have to be tested clinically.

## Supporting information

S1 Table Mutations in each isolate of SARS-CoV-2 in Africa in the early phase of COVID-19 epidemic. (XLS)

S2 Table Regional distribution of S614G, ORF1ab P4715L and ORF1ab RdRp in Africa. (XLSX)

S3 Table List of Acknowledgment of authors, originating and submitting laboratories of SARS-CoV-2 sequences on GISAID. (XLSX)

S4 Table Regional distribution of common and unique mutation of SARS-CoV-2 in Africa. (XLSX)

S5 Table Updated mutations in each isolate of SARS-CoV-2 in Africa. (XLSX)

S1 Fig In-country analysis of common and unique nucleotide variations in SARS-CoV-2 in African. Venn diagrams represent shared and unique nucleotide variations in SARS-CoV-2 in each country in Africa (only countries with available genome sequences were analyzed); (a) Benin, (b) Gambia, (c) Sierra Leone, (d) Uganda, (e) South Africa, (f) Kenya, (g) Ghana, (h) Morocco, (i) Senegal, (j) DRC, (k) Mali, (l) Nigeria, (m) Egypt, (n) Algeria, (o) Tunisia. Numbers in intersections represent number of shared mutations and numbers in non-intersected portions represent number of unique mutations in isolates in-country.
(TIF)
