# Supplementary figures and images for: Genomic and epidemiological characteristics of SARS-CoV-2 in Africa
Source: PLoS Negl Trop Dis. 2021 Apr 26;15(4):e0009335. doi: 10.1371/journal.pntd.0009335 (PMC8101992; doi:10.1371/journal.pntd.0009335)

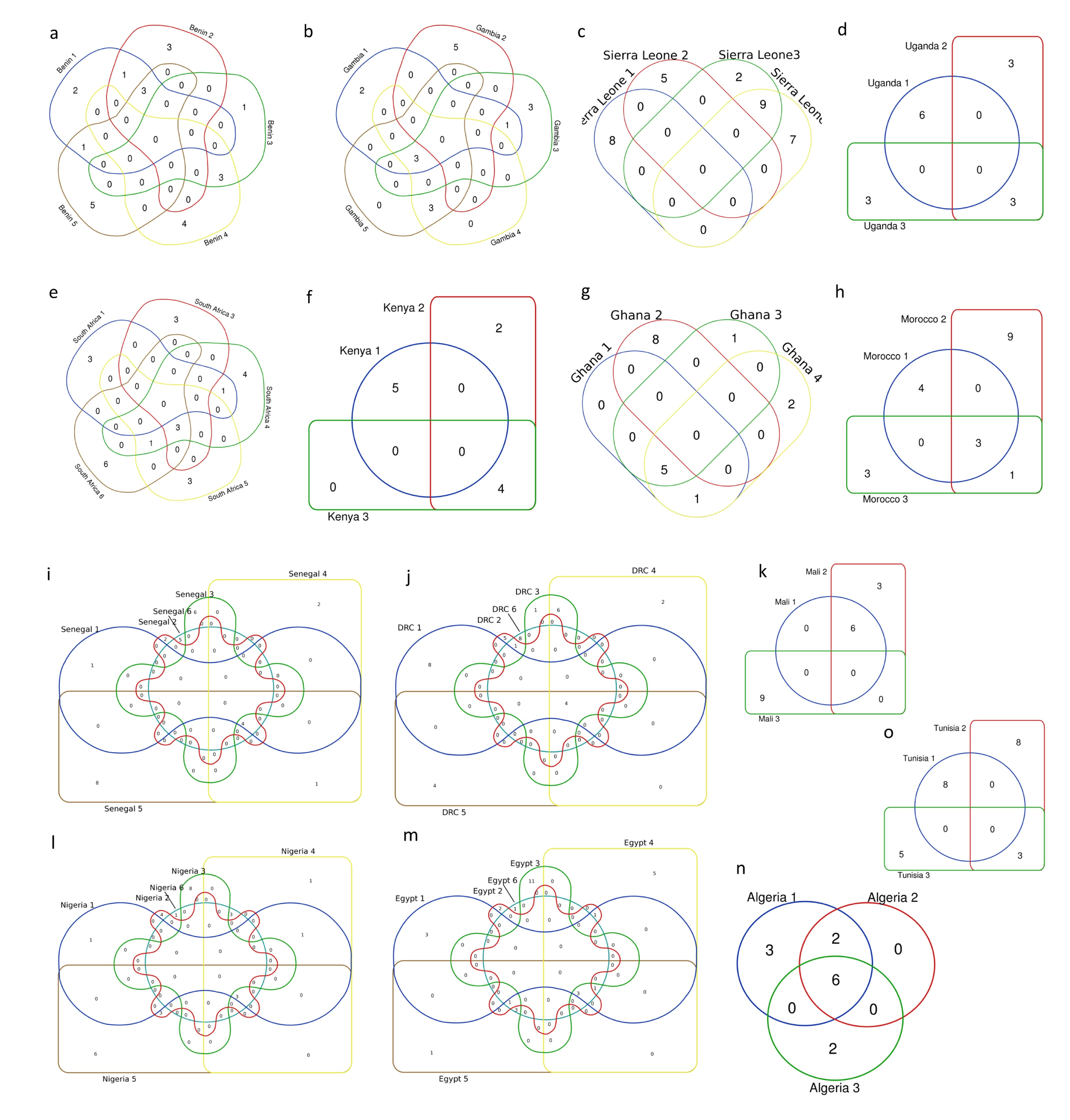

Supplement: S1 Fig — Venn diagrams represent shared and unique nucleotide variations in SARS-CoV-2 in each country in Africa (only countries with available genome sequences were analyzed); (a) Benin, (b) Gambia, (c) Sierra Leone, (d) Uganda, (e) South Africa, (f) Kenya, (g) Ghana, (h) Morocco, (i) Senegal, (j) DRC, (k) Mali, (l) Nigeria, (m) Egypt, (n) Algeria, (o) Tunisia. Numbers in intersections represent number of shared mutations and numbers in non-intersected portions represent number of unique mutations in isolates in-country. (TIF) [file pntd.0009335.s006.tif]
